# Identification of Ellagic Acid as a Natural GPR35 Agonist for Ulcerative Colitis Therapy

**DOI:** 10.3390/biom16030434

**Published:** 2026-03-13

**Authors:** Haichao Liu, Le Yang, Xiaoxu Ma, Guanying Wang, Dongxue Wang, Xiaokang Liu, Zhenwei Li, Dean Guo

**Affiliations:** 1 Guangzhou University of Chinese Medicine, Guangzhong 510006, China; liuhaichao1008@zidd.ac.cn (H.L.); yangle1310@zidd.ac.cn (L.Y.); maxiaoxu799@zidd.ac.cn (X.M.); wangguanying792@zidd.ac.cn (G.W.); 2 Zhongshan Institute for Drug Discovery, Shanghai Institute of Materia Medica, Chinese Academy of Sciences, Zhongshan 528400, China; wangdongxue0395@zidd.ac.cn (D.W.); liuxiaokang0583@zidd.ac.cn (X.L.); 3 Shanghai Academy of International Standardization for Traditional Chinese Medicine, Shanghai University of Traditional Chinese Medicine, Shanghai 201203, China

**Keywords:** GPR35, NanoBiT assay, ellagic acid, Ulcerative Colitis, intestinal epithelial repair

## Abstract

The escalating global burden of Ulcerative Colitis (UC) underscores the urgent need for novel therapeutic strategies. Although dietary modulation is known to influence UC progression, the specific molecular mediators remain largely undefined. Recently, the G protein coupled receptor 35 (GPR35) has emerged as a promising target for maintaining gut homeostasis and promoting intestinal epithelium repair. Yet, whether the therapeutic benefits of dietary polyphenols are mediated through the direct activation of GPR35 remains unexplored. Here, the NanoLuc Binary Technology (NanoBiT) assay was first used to identify the potential GPR35 agonist from a library of 30 natural polyphenolic compounds. We discovered Ellagic acid (EA), a natural polyphenol abundant in fruits and nuts, as the potent GPR35 agonist owing to its most potent agonistic effect. The dose-dependent effect was further confirmed by both NanoBiT and Bret assay. Then, the binding site of the ligand-receptor complex was predicted via molecular docking, and key interactions were validated by site-directed mutagenesis. The results indicated the key binding site of the complex was Gln93, Arg100, Arg151, Phe163 and Ser262. And the conformation of the complex was verified stable by the molecular dynamics simulation. The bioactivity of EA was then evaluated in vivo. And the in vivo experiment indicated that EA alleviated the symptoms of UC. In addition, complementary in vitro assays, including a wound healing (scratch) assay and an SRB proliferation assay, were employed to investigate its effect on intestinal epithelial repair. The in vitro experiment demonstrated that EA enhanced the migration and proliferation of human colonic epithelial cells, an effect that was specifically abolished by the GPR35 antagonist CID2745687, indicating the key role GPR35 played in the intestinal repair. Collectively, our study demonstrates that the natural polyphenolic compound EA promotes epithelial healing and ameliorates colitis by acting as a GPR35 agonist.

## 1. Introduction

As a chronic and relapsing type of inflammatory bowel disease (IBD), UC is characterized by continuous and diffuse inflammation of the colonic mucosa. Common clinical symptoms primarily include abdominal pain, diarrhea and bloody stools [1]. Epidemiologically, the incidence of UC has been rising steadily, rendering it a growing global health challenge [2]. Current consensus suggests that the etiology of UC involves a complex interplay of genetic susceptibility, immune dysregulation, alterations in the gut microenvironment, infectious agents, and dietary factors. However, despite extensive research, a comprehensive understanding of its precise pathogenesis remains elusive [3].Current therapeutic strategies of UC, including 5-aminosalicylic acid agents (5-ASA), corticosteroids, immunosuppressants, biologics, and Janus kinase (JAK) inhibitors, are deployed according to disease stage [4]. Yet, due to limitations such as significant side effects, high costs, and the potential for drug dependency or loss of response, there is an urgent need for alternative or complementary therapeutic approaches [5].

As the largest and most versatile family of cell surface receptors in the human genome, G Protein-Coupled Receptors (GPCRs) play a fundamental role as signaling conduits, translating extracellular stimuli into intracellular responses [6]. Given that approximately 37% of currently approved drugs target GPCRs, this receptor family represents a promising avenue for the discovery of novel therapeutics, including those derived from natural products [7]. GPR35, an orphan GPCR highly expressed in the gastrointestinal tract—particularly in intestinal epithelial cells (IECs)—has been implicated in the maintenance of gastrointestinal homeostasis [8]. Genetic studies have indicated that the T108M coding variant in the GPR35 gene, which results in a hyperactive receptor, correlates with increased susceptibility to UC, thereby identifying GPR35 as a potential risk gene for IBD [9]. Although several molecules, such as zaprinast, lodoxamide, pamoic acid and compound1, have been identified as potential GPR35 agonists, the identification of specific ligands derived from natural products remains largely unexplored [10].

There are many methods in drug screening of GPCR, including Cyclic adenosine monophosphate (cAMP) assay, calcium assay, Fluorescent Resonance Energy Transfer (FRET), Bioluminescence resonance energy transfer (BRET), β-arrestin recruitment assay, NanoBiT assay and so on [11]. Among these, BRET and NanoBiT assays have gained prominence in ligand discovery due to their favorable signal-to-noise ratios and high sensitivity [12]. The specific recruitment and activation of intracellular G proteins and β-arrestins are triggered by the conformational changes that GPCRs undergo upon ligand binding. Recent advances in structural biology, such as the resolution of the GPCR-Gα13 complex structure via cryo-electron microscopy, have provided a critical foundation for the rational screening of GPR35 ligands [13]. In addition, computational biology approaches, most notably molecular docking and molecular dynamics simulations, are also widely applied in binding site prediction and structure-based drug design [14,15]. For a known active molecule, molecular docking can offer insights into the microscopic mechanisms of ligand-receptor interactions [16]. However, it is important to note that the accuracy of molecular docking is directly tied to the quality of the target protein and the accuracy of the docking algorithm. Therefore, to ensure the reliability and biological relevance of the docking outcomes, subsequent validation is often required by integrating experimental methods, such as site-directed mutations [17,18]. In contrast to static docking, molecular dynamics simulations can reveal the conformational dynamics of receptor-ligand complexes through nanosecond or longer-scale dynamic modeling. By building upon docking results, molecular dynamics simulations are used to verify the stability and accuracy of docked conformations, and enhance the reliability of docking models and molecular recognition mechanisms [19]. This integrated computational-experimental strategy has the potential to accelerate development timelines and reduce costs, playing an increasingly central role in novel therapeutic agent discovery [20].

Accumulating evidence suggests that dietary intake plays a critical role in UC management potentially by modulating gut microbiota composition, epithelial barrier function, and mucosal immunity [21]. For instance, in murine colitis models, administration of Porphyra tenera was shown to ameliorate disease severity, an effect potentially mediated by the attenuation of intestinal inflammation and the improvement of microbiota dysbiosis [22]. Similarly, a case–control study suggested that higher dietary intake of vitamin C, vegetables, and retinol is associated with a reduced risk of UC [23]. Collectively, these findings highlight dietary intervention as a vital approach for UC prevention and management. Polyphenols, widely distributed in fruits, vegetables, grains, tea, and coffee, represent common bioactive constituents of the human diet [24]. Owing to their well-documented antioxidant and anti-inflammatory activities, Polyphenols have recently gained increasing attention for their potential in treating UC. For example, hydroxytyrosol, a polyphenol abundant in olive oil, has been reported to exert protective effects on the intestinal tract, possibly through the activation of the PI3K/Akt-Nrf2 signaling pathway, thereby attenuating oxidative stress [25]. Likewise, Forsythia suspensa polyphenols were found to alleviate symptoms in UC mice, potentially by inhibiting macrophage polarization toward the pro-inflammatory M1 phenotype [26]. Beyond their direct antioxidant and anti-inflammatory effects, the role of polyphenolic compounds in modulating the gut microbiota has also been increasingly recognized. Epigallocatechin-3-gallate (EGCG), the major bioactive component of green tea polyphenols, has been demonstrated to alleviate colitis symptoms in mice, an effect that may be linked to modulation of the gut microbiota [27]. Despite their broad bioactivity, the precise molecular targets of most polyphenols remain poorly defined, which hinders the full realization of their therapeutic potential in UC.

Diet plays an increasingly important role in the management of UC, and polyphenolic compounds are among the most common bioactive constituents in the diet. GPR35 has been identified as a susceptibility gene for UC and is known to be critical for maintaining intestinal homeostasis. However, no studies to date have systematically explored whether dietary polyphenols can exert therapeutic effects in UC through GPR35 activation. To address this gap, we first conducted a literature search and selected 30 common and representative food-derived polyphenolic compounds (Supplementary Table S1) for screening potential GPR35 agonists. For the candidate compound identified, we then employed a combination of molecular docking, site-directed mutagenesis, and molecular dynamics simulations to investigate its interaction mode, key binding sites, and binding stability with GPR35. Furthermore, we evaluated the therapeutic efficacy of this candidate compound in a UC model and used the GPR35-specific antagonist CID2745687 [28] to determine whether its effects are GPR35-dependent. These investigations aim to elucidate the potential mechanism by which dietary polyphenols may regulate UC pathology via GPR35 activation, offering new insights into diet-based therapeutic strategies.

## 2. Materials and Methods

### 2.1. Materials

All phenolic compounds (purity ≥ 98%) was purchased from Nature Standard Co., Ltd. (Shanghai, China). The HEK-293T cells were obtained from ATCC (ATCC # CRL-11268), and the normal colonic epithelial cell line NCM460 was provided from Guangzhou Xinyuan Biotechnology Co., Ltd. (Guangzhou, China). The Dulbecco’s modified Eagle’s medium (DMEM) was purchased from Thermo Fisher Scientific (Waltham, MA, USA), and the fetal bovine serum (FBS) was sourced from Gemini Bio (West Sacramento, CA, USA). The liposomal reagent, CTZ-400a and penicillin–streptomycin was provided from Yeasen Biotechnology Co., Ltd. (Shanghai, China). The NanoLuc substrate (furimazine) and CID2745687 were provided by Topscience Biochemical Technology Co., Ltd. (Shanghai, China). Dextran sulfate sodium (DSS) was sourced from Dalian Meilun Biotechnology Co., Ltd. (Dalian, China). Hematoxylin and eosin (HE) solution and Carboxymethyl Cellulose-Na (CMC-Na) was obtained from Beijing Solarbio Science & Technology Co., Ltd. (Beijing, China). Primary antibody against ZO-1 (Zonula Occludens-1) was obtained from Proteintech Group, Inc. (Wuhan, China). Sulforhodamine B (SRB) was purchased from Sigma-Aldrich (St. Louis, MO, USA).

### 2.2. NanoBiT Assay

We engineered a pbit vector encoding full-length human GPR35 (residues 1–309) with an N-terminal FLAG tag and a C-terminal LgBiT and encoding full-length β-arrestin2 with an N-terminal SmBiT. Eight site-directed mutations (Q93A, L97A, R100A, R151A, F163A, H168A, R240A, and S262A) were introduced into the construct. HEK-293T cells were grown using DMEM plus 10% FBS, under conditions of 37 °C and 5% CO_2_ in a humidified environment. For NanoBiT assay, a cell suspension was prepared at a concentration of 3.5 × 10^5^ cells per well and subsequently inoculated into 6-well plates. After 24 h, transfections were performed using 7 μL of liposomal reagent per well, with each well receiving 1.5 μg of either wild-type or mutant GPR35 plasmid together with 1.5 μg of β-arrestin2-encoding plasmid. Following transfection, cells were harvested at the 48 h time point and then resuspended in Tyrode’s buffer (Coolaber, Beijing, China). In the screening assay, each well of a 96-well plate received 70 μL of cell suspension, after which 20 μL of ligand solution was added (pre-diluted to fixed concentrations in Tyrode’s buffer) and 10 μL of diluted furimazine. Lodoxamide and phosphate-buffered saline (PBS) served as positive and negative controls, respectively. Luminescence was recorded at 25 °C using a BioTek plate reader, with measurements taken every 30 s over a 15 min interval. For concentration–response experiments, ligands were serially diluted in Tyrode’s buffer and applied as described above. All signals were normalized to vehicle-only controls. Following independent replication in at least three separate experiments, each performed in duplicate, the data (presented as mean ± SEM) were subsequently analyzed with GraphPad Prism 9.0.

### 2.3. Bret Assay

We engineered a pcDNA vector encoding full-length human GPR35 (residues 1–309) and mouse GPR35 with an N-terminal FLAG tag and a C-terminal LgBiT. HEK-293T cells were grown using DMEM plus 10% FBS, under conditions of 37 °C and 5% CO_2_ in a humidified environment. For BRET assays, a cell suspension was prepared at a concentration of 3.5 × 10^5^ cells per well and subsequently inoculated into 6-well plates. After 24 h, transfections were performed using 7 μL of liposomal reagent per well. Each well received 0.75 μg of wild-type GPR35 plasmid, together with 0.75 μg each of plasmids encoding Gα_13_, Gβ_1_-SmBiT and Gγ_1_. After 48 h, cells were collected and resuspended in Tyrode’s buffer (Coolaber, China). For the assay, each well of a 96-well plate received 70 μL of cell suspension, after which 20 μL of ligand solution was added (pre-diluted to fixed concentrations in Tyrode’s buffer) with 10 μL of diluted CTZ-400a. Subsequent steps were performed as described for the NanoBiT assay.

### 2.4. Molecular Docking

ChemBioDraw Ultra 14.0 was employed to generate the 2D structure of EA, which was subsequently rendered into a 3D model with ChemBio3D Ultra 14.0. AutoDock Tools 1.5.6 was used to convert the resulting structure, which had been saved in mol2 format, into the pdbqt format. The Protein Data Bank (RCSB PDB, http://www.rcsb.org) was the source from which the molecular structure of GPR35 (PDB ID: 8H8J) was obtained in PDB format. The structure was prepared by removing water molecules and existing ligands, adding hydrogen atoms, and performing other necessary adjustments in PyMOL 2.6.0. It was then converted to pdbqt format using AutoDock Tools 1.5.6. In AutoDock Tools 1.5.6, map files were generated and a grid box was defined to encompass the putative binding site, with its center and dimensions adjusted to appropriately cover the active pocket. AutoDock Vina, using the Lamarckian genetic algorithm, was employed to perform the molecular docking. The output docking poses were ranked according to their calculated binding energies, based on the energy scores, the conformation with the most favorable score was designated as representing the optimal binding mode. The highest-scoring pose was visualized using PyMOL.

### 2.5. Molecular Dynamics Stimulation

Molecular dynamics simulations were performed using GROMACS. Initial structures were prepared by converting protein PDB files to gro format with the AMBER99SB-ILDN force field applied to the protein. Small-molecule ligands were parameterized with the GAFF force field; hydrogen atoms were added and RESP partial charges were derived using Gaussian 16W. Each protein–ligand complex was solvated in a periodic box with TIP3P water molecules, maintaining a minimum distance of 1.2 nm between the protein and the box edges. The system was neutralized by adding Na^+^ ions. Energy minimization was carried out using the steepest-descent algorithm. A 1.0 nm cutoff was used for truncating short-range van der Waals and electrostatic interactions, whereas the particle-mesh Ewald method was employed to handle long-range electrostatics. Following the application of the LINCS algorithm to constrain hydrogen bonds, the system underwent equilibration. This involved a 100 ps NVT simulation prior to a 100 ps NPT simulation conducted at 300 K and 1 bar. Subsequently, a production-phase simulation of 200 ns was carried out under NPT conditions using a 2 fs time step. During this simulation, coordinates, velocities, and energies were recorded at 10 ps intervals for future analysis.

### 2.6. Animals Experiment

C57BL/6J mice were selected for our in vivo study due to their higher susceptibility to DSS compared to other strains. These mice develop robust and reproducible clinical symptoms (e.g., weight loss, diarrhea, and bloody stools) and histological features that closely mimic human UC. Eighteen male C57BL/6 mice (6 weeks old, 20–22 g) were provided by Beijing Vital River Laboratory Animal Technology Co., Ltd. (Beijing, China). The animal experimental procedure was approved by the Institutional Animal Care and Use Committee (IACUC) of the Zhongshan Institute for Drug Discovery, Shanghai Institute of Materia Medica, Chinese Academy of Sciences (IACUC No. 2024-12-GDA-01). All animals were housed under specific pathogen-free conditions. The environmental parameters were strictly controlled as follows: temperature, 23 ± 3 °C; relative humidity, 55 ± 15%; and a 12 h light/12 h dark photoperiod. The mice were kept three to a cage and provided with food and water ad libitum. In this experiment, we employed DSS to induce mouse model of UC. DSS is a powerful tool for studying animal colitis, as it can rapidly induce pathological features similar to those of human colitis, including intestinal mucosal congestion, edema, ulceration, inflammatory cell infiltration, as well as symptoms such as diarrhea and hematochezia [29,30]. After a 7-day acclimation period, they were randomly assigned to the following groups: Control, Model, and EA, with six mice in each group. The Control group received distilled water only. To induce colitis, both the Model and EA groups were administered distilled water containing 3% (*w*/*v*) DSS for seven consecutive days [31]. Beginning on the same day as DSS exposure, mice in the EA group were treated daily by oral gavage with EA (60 mg/kg) suspended in 0.5% CMC-Na for 8 days. Control and Model mice received an equal volume of vehicle (0.5% CMC-Na). All animals were maintained on a standard diet. We detected the disease activity by assessing body weight, stool consistency, and the presence of fecal blood each day at a consistent time, according to an established disease activity index (DAI) scoring system (Supplementary Table S2). Subsequently, the average of the three parameter scores was calculated as the final DAI score [32,33]. On day 9, mice were anesthetized by intraperitoneal injection of Zoletil 50 (30 mg/kg). Following collection from the abdominal aorta into heparin-coated tubes, blood was centrifuged to obtain the plasma component. Following cervical dislocation, the entire colon was removed and its length recorded. The isolated tissue was then gently rinsed with ice-cold saline. Finally, the segment of the distal colon was fixed in 4% paraformaldehyde for subsequent HE-staining and immunofluorescence analysis. NoteGiven the complexity of GPR35 in vivo mechanisms and the lack of an established positive control compound for UC research targeting GPR35, no positive control group was included. Target specificity will be further verified in future work using GPR35 knockout mice. Notably, additionally, since this study focused on assessing the therapeutic potential of ellagic acid rather than benchmarking it against clinical standards, a comparative positive control was deemed unnecessary.

### 2.7. H&E Staining and Immunofluorescence Analysis

Fresh tissue samples were fixed in fixative solution for a minimum of 24 h. Following fixation, the specimens were trimmed, dehydrated through a graded ethanol series, cleared in xylene, and embedded in paraffin. Sections were cut at a thickness of 4 μm using a microtome. For histological analysis, deparaffinized sections were stained with H&E staining, mounted with neutral balsam, and scanned in their entirety at 20× magnification using an Olympus SLIDEVIEW VS200 slide scanner (Olympus Corporation, Hachioji, Tokyo, Japan). The resulting whole-slide images were evaluated based on a standardized histopathological scoring system shown in Supplementary Table S3 [34]. For immunofluorescence staining, following pretreatment involving antigen retrieval and blocking with Bovine Serum Albumin (BSA) for 30 min, the sections were incubated overnight at 4 °C in the dark with an primary antibody (ZO-1,diluted 1:500). Subsequently, a 1 h incubation with a 488-conjugated secondary antibody, the samples were nuclear-stained with DAPI and the prepared slides were mounted with an anti-fade medium. High-resolution images were acquired using a Leica STELLARIS STED confocal microscope (Leica Microsystems, Wetzlar, Germany). We performed the quantitative analysis of ZO-1 mean fluorescence density using ImageJ software (ver. 1.5.7 from the National Institutes of Health, USA).

### 2.8. Wound Healing Assay

NCM460 cells are internationally recognized as a normal human colonic epithelial cell line. Due to their retention of the primary biological characteristics of normal intestinal epithelial cells, they have become a classic in vitro model for studying intestinal physiology, pathology, and the intestinal epithelial barrier function. Under standard incubator conditions (37 °C, 5% CO_2_, humidified), NCM460 cells were cultured in DMEM containing 10% fetal bovine serum (FBS) and 1% penicillin-streptomycin. For the wound healing assay, we seeded cells into 6-well plates at 5 × 10^5^ cells per well and cultured them until they achieved 90–100% confluence. A sterile 100 µL pipette tip was guided vertically using a straightedge to generate three parallel, uniform scratches in each well. Once detached cells were removed by washing with PBS, the remaining cells received the following treatments in serum-free medium: vehicle control (containing DMSO), 15 µM EA, 10 µM CID2745687, or a combination of both 10 µM CID2745687 and 15 µM EA. Wound images of each group were acquired at 0 h and 24 h using a Nikon microscope (Nikon, Tokyo, Japan) at 20× magnification, and then wound closure was quantified using ImageJ software.

### 2.9. SRB Assay

At each designated time point (6, 24, 48, and 72 h), the culture medium was first aspirated. It was then replaced with 50 µL of trichloroacetic acid (TCA) solution at a final concentration of 50% to fix cells. After incubation at 4 °C for 1 h, the fixed cells were washed at least five times with distilled water and allowed to dry completely. For staining, a freshly prepared solution of 0.4% SRB in 1% acetic acid was added to all wells, after which the plates were kept at room temperature, protected from light, for a 15 min incubation period. Following incubation, the staining solution was removed, and the microtiter plates were washed with 1% acetic acid to eliminate any unbound dye. The protein-bound SRB dye remaining in the wells was then solubilized using a 10 mM Tris base solution. Finally, the absorbance was measured at a wavelength (λ) of 510 nm using a BioTEK plate reader (Agilent Technologies, Santa Clara, CA, USA).

### 2.10. Statistical Analysis

Data analysis was conducted with SPSS software (version 25.0). Post hoc pairwise comparisons, conducted following one-way ANOVA, employed either the LSD test when the assumption of homogeneity of variance was met, or the Games-Howell test when it was violated. Data are presented as mean ± SEM, with between-group differences quantified using 95% confidence intervals. Statistical significance was assigned to findings where the two-sided *p*-value fell below 0.05.

## 3. Results

### 3.1. Agonist Screening of GPR35 Among Dietary Polyphenols Using the NanoBiT Assay

**Figure 1:**
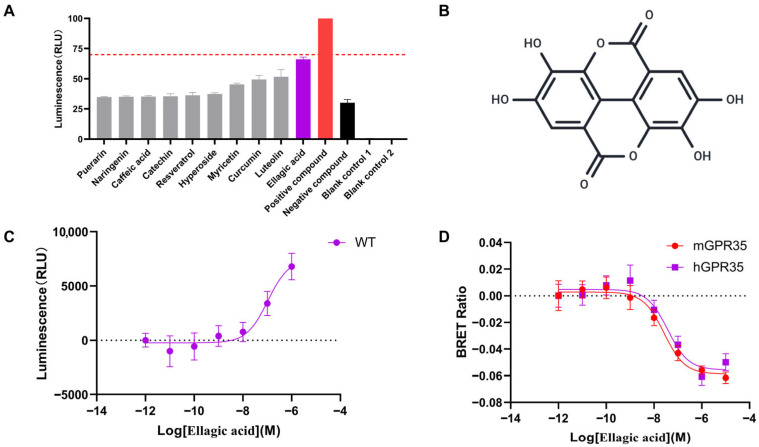
Agonist screening of GPR35 among dietary polyphenols using the NanoBiT β-arrestin2 recruitment assay. (**A**) Efficacy profile of 30 dietary polyphenols on human GPR35-mediated β-arrestin2 recruitment. Compounds were screened at a concentration of 10 μM. Data are normalized to the response of Lodoxamide (10 μM), a known potent GPR35 agonist serving as the positive control in this study, which is defined as 100% efficacy (red bar). The *Y*-axis represents ‘% of Lodoxamide response’. The purple bar indicates EA, which exhibited the highest agonist activity among the tested polyphenols. (**B**) Chemical structure of EA. (**C**) Dose–response curves of EA-induced β-arrestin2 recruitment at human GPR35. Cells were treated with EA at concentrations ranging from 10^−6^ M to 10^−12^ M via 10-fold serial dilutions. Specific β-arrestin2 recruitment responses were calculated by subtracting the ligand-free baseline (vehicle control) from the raw data prior to curve fitting. (**D**) Species-dependent agonist activity of EA. Comparative dose–response profiles of EA at human (purple curve) and mouse (red curve) GPR35 orthologs were generated using the same concentration range and dilution strategy as in panel (**C**). Data are all presented as mean ± SEM (*n* = 3 independent experiments performed in triplicate). Abbreviations: EA, Ellagic Acid; GPR35, G protein-coupled receptor 35; NanoBiT, NanoLuc Binary Technology; SEM, standard error of the mean.

In an effort to discover novel natural agents capable of effectively activating GPR35, we performed a drug screening using the NanoBiT assay on 30 common polyphenol primarily sourced from nuts, vegetables, and fruits. β-arrestin proteins and G proteins are major downstream effector proteins of GPCRs, and their recruitment levels can indirectly reflect the activation status of the receptor. As shown in Figure 1A, we examined the recruitment of β-arrestin2 by these 30 compounds and found that EA exhibited the strongest activating effect (NanoBiT-based screening of all 30 polyphenolic compounds for GPR35 agonists was shown in Supplementary Figure S1). Moreover, through NanoBiT experiments, we demonstrated that the activation of hGPR35 by EA was concentration-dependent (Figure 1C). To further confirm the agonist activity of EA, we employed BRET technology, which detects G protein recruitment, and found that EA could recruit Gα13 in a concentration-dependent manner, suggesting its potential as a ligand for hGPR35. Since ligand-induced activation of GPR35 exhibits species-specific effects, we also evaluated the activation of mouse-derived GPR35 by EA. The results indicated that EA can also active mGPR35 in concentration-dependent manner, and the activation of both human and mouse GPR35 exhibited the comparable potency (Figure 1D). Herein, we demonstrated that the dietary polyphenol EA acts as a dose-dependent agonist of GPR35 in both human and murine systems.

### 3.2. Binding Mode and Key Interactions of EA with GPR35

**Figure 2:**
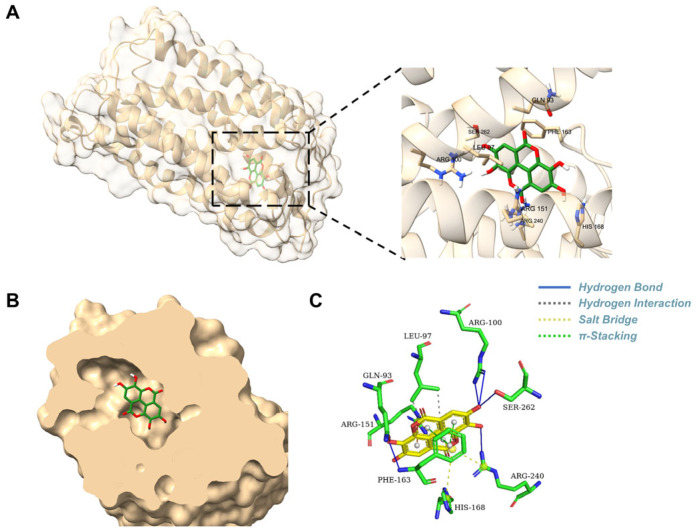
Molecular docking model of EA in the GPR35-binding site. (**A**) 3D docking pose of EA within the GPR35 binding site. The GPR35 structure is visualized as a yellow ribbon. An enlarged view illustrates EA’s positioning within the binding pocket. (**B**) Cross-section of the lodoxamide-binding pocket in GPR35. (**C**) Interaction model of GPR35 and EA.

This docking conformation exhibited a strong binding affinity, with a calculated energy of −8.709 kcal/mol. The surface model shown in Figure 2A,B demonstrated that the ligand is snugly embedded within a cavity on the protein surface, a configuration that is complementary to the ligand’s structure and favorable for stable binding. As shown in Figure 2C, five hydrogen bonds (including Gln93, Arg100, Phe163, Arg240, Ser262) were formed between the ligand and the protein’s amino acid residues, according to the three-dimensional structural analysis. Specifically, a hydrogen bond with a length of 2.29 Å was observed between the ligand and the residue of Gln93 and Arg100, indicating that these residues constitute key binding sites. The two-dimensional interaction diagram further illustrated that van der Waals forces from residues of Leu97 surround the ligand, which are essential for stabilizing the binding site. Additionally, two π-interactions (e.g., π-cation, π-π stacking, etc.) were identified between the ligand and the residues Arg151 and Phe163, further influencing the binding affinity and specificity. Furthermore, the ligand participates in a stable electrostatic network by forming salt bridges with three key positively charged residues, including Arg151, His168, and Arg240. In summary, a relatively strong and specific interaction between the EA and the GPR35 was indicated by the molecular docking results.

### 3.3. Functional Analysis of Wild-Type and Mutant Receptors

**Figure 3:**
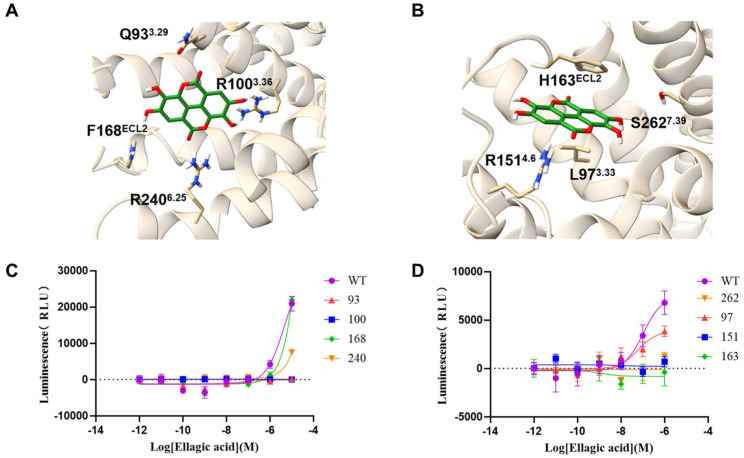
Functional analysis of wild-type and mutant receptors. (**A**) Model showing key hydrophobic residues (Q93, R100, F168 and R240) interact with EA in the binding pocket. (**B**) Model showing key hydrophobic residues (L97, R151, H163 and S262) interact with EA in the binding pocket. (**C**,**D**) NanoBiT assay showing the response of wild-type and mutant GPR35 receptors to EA.

To experimentally validate the predicted complex of EA binding to GPR35, the site-directed mutagenesis was performed followed by molecular docking. In site-directed mutations, the key amino acid residue was mutated to smaller residues (Ala or Gly), resulting in the loss of their original interactions. Therefore, the site-directed mutations (Q93A, L97A, R100A, R151A, F163A, H168A, R240A, S262A), with the key amino acid residues illustrated in Figure 3A,B, were generated and evaluated for receptor activation using a NanoBiT assay. As shown in Figure 3C,D, the Q93A, R100A, R151A, F163A and S262A mutations completely abolished EA-induced receptor activation compared with wild-type (WT) GPR35. The L97A and R240A mutants reduced the maximal activation efficacy (ECmax) of EA by approximately twofold, indicative of substantially diminished binding affinity. In contrast, the H168A mutant exhibited activation levels comparable to WT, suggesting that His168 is not a critical residue for EA binding to GPR35. Collectively, these results identify Gln93, Arg100, Arg151, Phe163 and Ser262 as critical residues for EA binding and activation of GPR35, while Leu97 and Arg240 appear to play auxiliary roles in the interaction. Our findings provide experimental support for the computationally predicted binding site and mode of action of EA on GPR35.

### 3.4. Molecular Dynamics Simulation of GPR35 and EA

**Figure 4:**
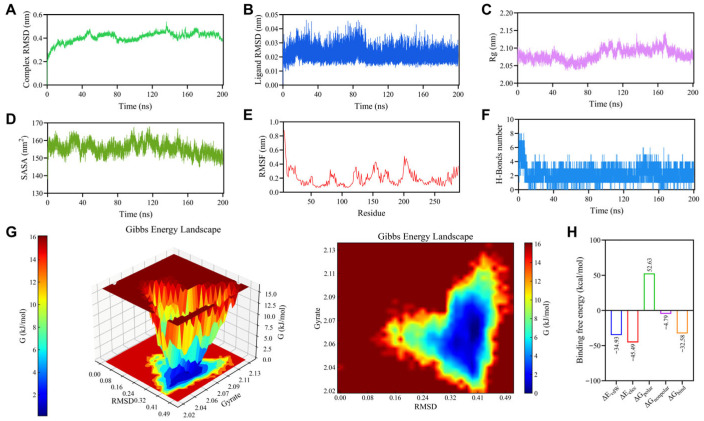
Analysis of 200 ns molecular dynamics simulations of the complex. (**A**) RMSD (complex). (**B**) RMSD (ligand). (**C**) Rg. (**D**) SASA. (**E**) RMSF. (**F**) Protein-ligand H-bonds. (**G**) Free energy landscape. (**H**) Binding free energy.

Molecular dynamics simulations provide a means to monitor, in real time, the binding stability and dynamic interactions within small molecule–protein complexes. As shown in Figure 4A,B, RMSD analysis revealed a rapid increase for the complex in the first 20 ns of simulation, after which the fluctuations diminished, suggesting the attainment of dynamic equilibrium. As shown in Figure 4C, the Rg value showing only minor oscillations over time without any systematic upward or downward trend, and the SASA values exhibiting slight oscillations over time but no abrupt changes, suggesting that a modest compaction or rearrangement of hydrophobic regions within the protein during the simulation may facilitate tighter ligand binding, thereby contributing to the maintenance of a stable configuration. To investigate the effect of ligand binding on the flexibility of protein residues, we calculated the RMSF of each amino acid over the entire simulation. As illustrated in Figure 4E, most residues exhibited fluctuations below 0.3 nm. Slightly higher fluctuations were observed in certain regions, predominantly at the N-/C-termini or loop regions, while the core structural regions and potential binding sites displayed restricted mobility. Hydrogen bonds represent strong non-covalent interactions, and their number was therefore used to assess the stability of the protein–ligand complex during the simulation. To further examine hydrogen-bond dynamics, we statistically analyzed intermolecular hydrogen bonds formed between the ligand and the protein over the 200 ns trajectory. As shown in Figure 4F, the system maintained approximately 2–4 hydrogen bonds throughout the simulation, with occasional higher values at certain time points, and no prolonged periods of complete absence were observed. This consistent presence of multiple hydrogen bonds suggests a stable binding interface. The free-energy landscape (FEL) projected onto the RMSD–Rg reaction coordinates revealed a single, well-defined low free-energy basin for the protein–ligand complex (Figure 4G). The global minimum was concentrated in the region corresponding to RMSD values of approximately 0.32–0.41 nm and Rg values of approximately 2.04–2.08 nm, indicating that the conformation corresponds to a state of relatively high stability. The binding between the protein and ligand was primarily driven by van der Waals and electrostatic forces, with the latter making a notably dominant contribution, as revealed by MM/PBSA binding free energy decomposition (Figure 4H). The final calculated binding free energy was −32.58 kcal/mol. All molecular dynamics simulation results collectively demonstrate that the GPR35–EA complex structure predicted by molecular docking exhibits robust stability.

### 3.5. EA Administration Ameliorates DSS-Induced Colitis in Mice

**Figure 5:**
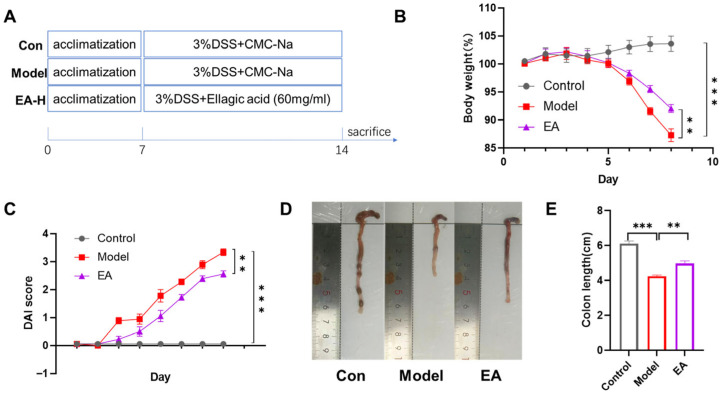
EA administration ameliorates DSS-induced colitis in mice. (**A**) Schematic diagram of the experimental design. (**B**) Time course of body weight. (**C**) Change in DAI score. (**D**) Representative colon images from each group. (**E**) Colon length of each group. Data are shown as mean ± SEM. ** *p* < 0.01, *** *p* < 0.001 versus Model group.

In this study, the therapeutic potential of EA for colitis was evaluated by employing a DSS-induced mouse model. This DSS-induced colitis model is widely employed in drug screening for UC treatment owing to its close pathological resemblance to human UC, including symptoms such as intestinal mucosal congestion, edema, ulceration, and inflammatory cell infiltration, as well as diarrhea and hematochezia [35]. As shown in Figure 5B,C, mice in the model group receiving 3% DSS in drinking water exhibited progressive weight loss (*** *p* < 0.001). Crucially, daily monitoring of the DAI score revealed a progressive and significant deterioration in the model group compared to control group (*** *p* < 0.001). In contrast, EA treatment markedly attenuated DSS-induced intestinal damage, as evidenced by a significant reduction in weight loss and a substantially lower DAI score in the model group compared to control group (** *p* < 0.01). Beyond these symptoms, colon shortening serves as a hallmark structural alteration in colitis. This phenomenon is associated with DSS-mediated disruption of the intestinal epithelial barrier, sustained inflammatory infiltration, and subsequent tissue fibrosis leading to colon wall contraction. As shown in Figure 5D,E, results demonstrated that colon length was significantly shorter in DSS-treated mice compared to the control group (*** *p* < 0.001). EA intervention significantly ameliorated this shortening, with colon length notably greater than that in the model group (** *p* < 0.01). Collectively, our results indicate that EA protects mice from DSS-induced colitis, markedly attenuating disease symptoms.

### 3.6. EA Preserves Colonic Structure and Barrier Function

**Figure 6:**
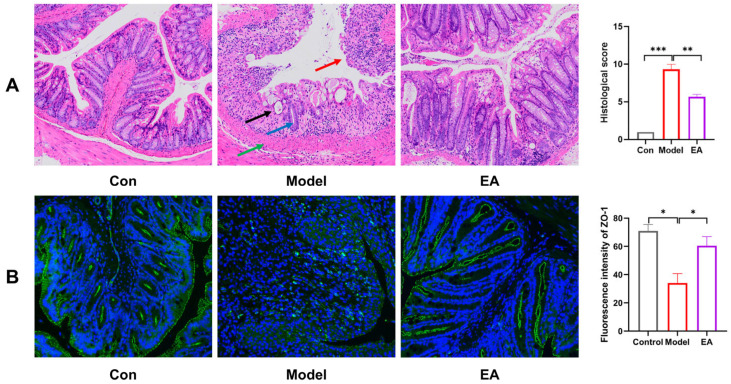
EA preserves colonic structure and barrier function. (**A**) H&E staining of each group (20×) and Histopathological assessment. (**B**) Immunofluorescence of ZO-1 in each group (200×). Data were presented as mean ± SEM. * *p* < 0.05, ** *p* < 0.01, *** *p* < 0.001 versus Model group.

The disruption of intestinal epithelial cells is often considered the primary cause of recurrent colitis, and the function of the biological barrier is closely linked to intestinal epithelial repair and tight junction proteins. First, histological changes in colonic tissue were assessed by H&E staining. As shown in Figure 6A, mice in the model group displayed pronounced pathological alterations compared with control group, including loss of intestinal epithelial cells (red arrow), disappearance of crypts (black arrow), widespread reduction in goblet cell numbers (blue arrow), and extensive inflammatory cell infiltration into the mucosal layer accompanied by muscularis mucosa thickening (green arrow). In contrast, treatment with EA significantly ameliorated colon damage, particularly promoting repair of the intestinal epithelium. Furthermore, the expression of ZO-1, a crucial tight junction protein that participates in maintaining tight junctions and barrier function between cells, was examined by immunofluorescence staining. As shown in Figure 6B, the results indicated that a significant decrease in ZO-1 expression was observed in the model group compared to the control group (* *p* < 0.05). EA treatment markedly restored the downregulated expression of ZO-1 (* *p* < 0.05). Together, these findings suggest that EA contributes to the preservation of intestinal barrier integrity by mitigating epithelial damage and enhancing the expression of key tight-junction proteins in a mouse model of colitis.

### 3.7. EA Promotes the Repair of the Intestinal Epithelium via Activating GPR35

**Figure 7:**
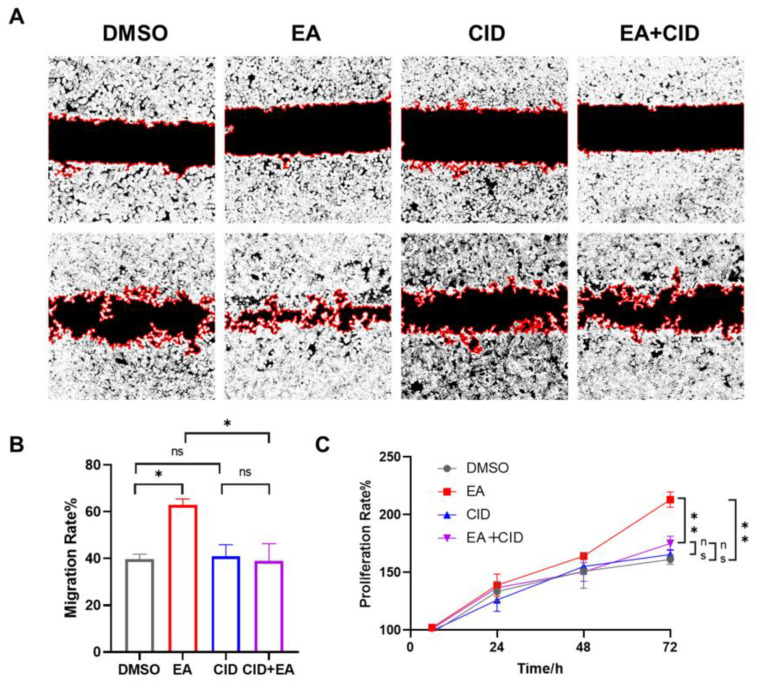
EA promotes the repair of the intestinal epithelium via activating GPR35. (**A**) Representative images of Wound Healing Assay. (**B**) The effect of EA on promoting migration of intestinal epithelium cells via GPR35. (**C**) The effect of EA on promoting proliferation of intestinal epithelium cells via GPR35. Data were presented as mean ± SEM. * *p* < 0.05, ** *p* < 0.01 versus Model group, ns > 0.05.

To investigate the role of GPR35 in epithelial repair, we then assessed the effect of EA on wound healing using a scratch assay. As shown in Figure 7B, compared to the DMSO treated control, EA significantly enhanced the migration of NCM460 cells over 24 h (* *p* < 0.05). The specific GPR35 antagonist CID2745687, when added alone, did not affect basal cell migration compared to the DMSO control. However, the pro-migratory effect of 20 µM EA was completely abolished by co-treatment with CID2745687. Consequently, the migration capacity in the EA + CID2745687 co-treatment group showed no significant difference from the CID2745687-alone group but was significantly lower than that in the EA-only group (* *p* < 0.05). The effect of EA on cell proliferation was further evaluated by using the SRB assay. As illustrated in Figure 7C, treatment with EA significantly increased the proliferation rate of NCM460 cells compared to the DMSO control (** *p* < 0.01). In contrast, the GPR35 antagonist CID2745687 alone had no measurable effect on proliferation. Notably, co-treatment with EA and CID2745687 resulted in a proliferation level comparable to that of the CID2745687-alone group and was significantly reduced relative to the EA-only group (** *p* < 0.01). Taken together, these results collectively demonstrate that EA enhances intestinal epithelial cell migration and proliferation through specific activation of GPR35, thereby revealing a key mechanism underlying its promotion of epithelial repair at a functional level.

## 4. Discussion

With the attribution to the Westernization of lifestyles, the prevalence in Asia, Latin America, and Africa experienced a 5 to 10-fold surge in UC incidence [36,37]. Unfortunately, current treatments for colitis are often associated with side effects, high costs, and a tendency for recurrence [38]. Consequently, the role of dietary intake in managing UC is attracting growing attention as a potential therapeutic method [39]. Polyphenols are plant-derived bioactive compounds commonly found in foods like vegetables, fruits, and nuts. Their demonstrated anti-inflammatory, antioxidant, and microbiota-regulating effects are driving their growing incorporation into functional food development [40]. Notably, bioactive components in breast milk and infant formula have been shown to activate GPR35, thereby underscoring its potential as a target for dietary interventions [41]. Moreover, GPR35 has emerged as a potential therapeutic target for UC treatment, with reported efficacy in promoting intestinal epithelial repair [42]. However, whether dietary components can act as ligands for GPR35 to ameliorate UC remains largely unexplored.

Thus, the NanoBiT assay, a method commonly used for GPCR ligand screening, was applied to screen polyphenolic compounds targeting GPR35 [43]. In this study, EA, a widely available dietary polyphenol, was identified as a novel and potent natural agonist of GPR35 from thirty dietary polyphenolic compounds. Interestingly, EA has found broad application in wound healing and tissue regeneration, but its effects on intestinal epithelial restitution remain unexplored [44,45]. So, the significant protective role of EA in DSS-induced colitis was highlighted in our vivo experiment, which was primarily mediated through the promotion of intestinal epithelial repair. Moreover, this protective effect was further verified to be dependent on GPR35 activation in the vitro studies, leading to enhanced migration and proliferation of intestinal epithelial cells. These findings link dietary polyphenols to GPCR-mediated intestinal epithelial protection, establishing a molecular basis for the health benefits of polyphenol-rich foods.

To comprehensively elucidate the molecular mechanism by which EA activates GPR35, we integrated computational modeling (in silico) with functional biochemical validation (in vitro). Initial molecular docking provided a static, high-affinity hypothesis for the EA–GPR35 complex, predicting a binding energy of −8.71 kcal/mol and identifying a specific network of hydrogen bonds and π-π interactions. However, static models cannot account for protein flexibility or solvent effects over time. To address this limitation and contextualize the strength of the docking prediction, we performed 200 ns molecular dynamics (MD) simulations. Collectively, these MD data demonstrate that the EA–GPR35 complex possesses the structural resilience required for biological signaling. To distinguish which interactions are essential for receptor activation, we employed site-directed mutation followed by NanoBit assays. This in vitro functional validation allowed us to map the computationally predicted interaction network onto biological efficacy. The complete abolition of EA-induced activity in Q93A, R100A, R151A, F163A, and S262A mutants confirms that the specific hydrogen bonds and π-π interactions predicted by docking and stabilized during MD are indispensable for triggering the conformational switch required for G-protein coupling. By synthesizing these three layers of evidence, we propose a refined mechanism of action for EA on GPR35. EA acts as a potent agonist by embedding into the transmembrane cavity, where it forms a dynamic yet rigid complex stabilized by electrostatic and van der Waals forces (validated by MD). Within this stable complex, specific interactions with Gln93, Arg100, Arg151, Phe163, and Ser262 act as the molecular switch, driving the receptor into an active conformation (validated by mutagenesis). This multi-dimensional characterization of the EA–GPR35 interaction, offering a solid structural foundation for the observed in vivo therapeutic effects and guiding the rational design of future GPR35-targeted therapeutics.

EA, a naturally occurring polyphenolic compound abundant in foods such as grape seeds, tea, and nuts, exhibits a wide range of biological activities, demonstrating considerable potential for health-promoting applications [46]. Aligning with the conclusions of Li, X et al. [47], our in vivo studies confirmed that EA can alleviate the symptoms of DSS induced colitis rats, including weight loss, DAI score and shortened colon length. And the damage to the intestinal epithelium and barrier function was significantly ameliorated following EA treatment. Recently, the therapeutic potential of EA in colitis has attracted growing interest. Research indicated that EA alleviates colitis symptoms by suppressing inflammatory responses and inhibiting NLRP3 inflammasome activation [48]. Furthermore, studies also reported that by scavenging reactive oxygen species, hydrogel-encapsulated EA nanoparticles augment their colonic retention and confer protection against DSS-induced colitis [49]. Notably, among its biological activities, the modulation of gut microbiota by EA has been extensively studied. Evidence suggests that Streptococcus abundance is closely associated with intestinal immune responses, and pretreatment with EA reduces the abundance of intestinal Streptococcus [50]. And treatment with EA restored Lactobacillus abundance while reducing the levels of Bacteroides and Escherichia coli, protecting the intestine from potentially harmful gut bacteria and fungi [51]. Although the repair of the intestinal epithelium is a critical therapeutic target in colitis, the role of EA in promoting intestinal epithelium repair remains underexplored.

Interestingly, it has been validated that the activation of GPR35 can promote intestinal epithelial repair. Studies indicate that GPR35 agonists promote intestinal epithelial repair through a mechanism involving Gi protein recruitment, activation of the ERK1/2 signaling pathway, and subsequent upregulation of fibronectin and integrin α5 expression [52]. During intestinal epithelial repair, IECs near the wound lose their polarity, and the microvilli on the cell surface disappear. Subsequently, they form protrusions that extend and migrate toward the damaged area, thereby facilitating wound closure [53]. The rapid proliferation of IECs replenishes the lost epithelial cells and likewise plays a key role in the overall repair process of the intestinal epithelium [54]. Our in vitro study, including wound healing assay and SRB assays, revealed that EA significantly enhanced intestinal epithelial cell migration and proliferation, but these promotive effects were blocked by the GPR35-specific inhibitor CID2745687, whereas the inhibitor alone showed no effect. In vitro studies demonstrate that EA promotes the proliferation and migration of intestinal epithelial cells, which is dependent on GPR35 activation. These findings reveal its potential in promoting epithelial repair and provide a new direction for developing functional foods targeting GPR35.

Several limitations of this study warrant consideration. First, our investigation primarily focused on the direct effects of EA, without fully disentangling whether the observed therapeutic outcomes are attributable to EA itself or its gut microbiota-derived metabolites, such as urolithins [55]. Given that urolithins have been independently confirmed to possess anti-UC activity and can modulate tryptophan metabolism [56,57]—and considering that tryptophan metabolites like kynurenic acid (KYNA) and 5-hydroxyindoleacetic acid (5-HIAA) are established endogenous ligands for GPR35 [58,59]—it remains to be explored whether EA exerts its efficacy partially through a urolithin–GPR35 axis. Future studies employing gut microbiota metabolomics will be essential to elucidate the extent to which EA’s therapeutic benefits are mediated by these microbial metabolites. Second, while our computational models provide strong predictive insights, the application of cryo-electron microscopy (cryo-EM) to resolve the structure of the EA–GPR35 complex would significantly strengthen the mechanistic interpretation of their interaction. The atomic-resolution structures obtained via cryo-EM would serve as a precise structural blueprint for the rational design and targeted modification of EA derivatives to enhance potency and selectivity. Third, given the lack of a mechanism-matched gold standard for in vivo studies, and because the mechanism of action of the clinical drug 5-ASA (broad-spectrum anti-inflammatory) does not align with the core hypothesis of this study (specific activation of GPR35), a conventional drug control was not included. In future studies, with the establishment of GPR35 genetically engineered mouse models, we will further refine the experimental design. Finally, to further explore the target spectrum of EA, future research could employ high-throughput screening assays, such as Presto-salsa, to investigate whether EA can activate other GPCRs. This would help map the full landscape of EA’s polypharmacology in the context of intestinal homeostasis.

## 5. Conclusions

In summary, EA was identified as a natural agonist of GPR35 from 30 polyphenolic compound via NanoBiT assay. EA is demonstrated to activate both human and murine GPR35 with equivalent potency, leading to the recruitment of Gα13 and β-arrestin2. The binding mode and key interaction sites of EA with GPR35 were predicted by molecular docking and substantiated through site-directed mutagenesis. Residues Q93A, R100A, F163A and S262A, which form hydrogen bonds with EA, were shown to play critical roles in receptor activation, along with the hydrophobic contribution of Leu97. Additional support was provided by Arg240 (hydrogen bond) and Arg151 (salt bridge). These predictions were further validated by molecular dynamics simulations, confirming the stable binding of EA to GPR35. Functionally, EA was found to alleviate symptoms in a murine colitis model, consistent with its known anti-inflammatory, antioxidant, and microbiota-modulating properties. Notably, a previously unreported role of EA in promoting intestinal epithelial migration and proliferation was uncovered in vitro. This pro-repair effect was reversed by the GPR35 antagonist CID2745687, underscoring the specificity of EA action through GPR35 activation. Collectively, these results highlight the protective role of EA in colitis and provide a mechanistic rationale for the development of EA-rich foods as potential dietary interventions for intestinal disorders.

However, whether EA exerts its anti-colitis effects synergistically with its intestinal metabolites remains unclear, necessitating further investigation via gut microbial metabolomics. Moreover, elucidating the precise binding mode between EA and GPR35 demands validation using advanced structural biology approaches, particularly cryo-electron microscopy. Additionally, given the potential for off-target effects, definitive validation using Gpr35-knockout mice is indispensable. Finally, comprehensive target screening assays will be crucial to map the full spectrum of receptors potentially activated by EA.

## Data Availability

The original contributions presented in the study are included in the article/Supplementary Materials, further inquiries can be directed to the corresponding authors.

## Supplementary Materials

The following supporting information can be downloaded at: https://www.mdpi.com/article/10.3390/biom16030434/s1, Figure S1: NanoBiT-based screening of 30 polyphenolic compounds for GPR35 agonists. Table S1: 30 Polyphenolic compounds. Table S2: Disease activity index (DAI). Table S3: Histopathological Score.

## Abbreviations

The following abbreviations are used in this manuscript:

UC	Ulcerative Colitis
EA	Ellagic Acid
GPR35	G Protein-Coupled Receptor 35
ZO-1	Zonula Occludens-1
GPCRs	G Protein-Coupled Receptors
IECs	Intestinal Epithelial Cells
