# Supplementary material for: Identification of Ellagic Acid as a Natural GPR35 Agonist for Ulcerative Colitis Therapy
Source: Biomolecules. 2026 Mar 13;16(3):434. doi: 10.3390/biom16030434 (PMC13024377; doi:10.3390/biom16030434)
Supplement: Supplementary file 1 [file biomolecules-16-00434-s001.zip › biomolecules-4152508-supplementary-1.pdf]

Figure S1. Nanobit-based screening of 30 polyphenolic compounds for GPR35 agonists

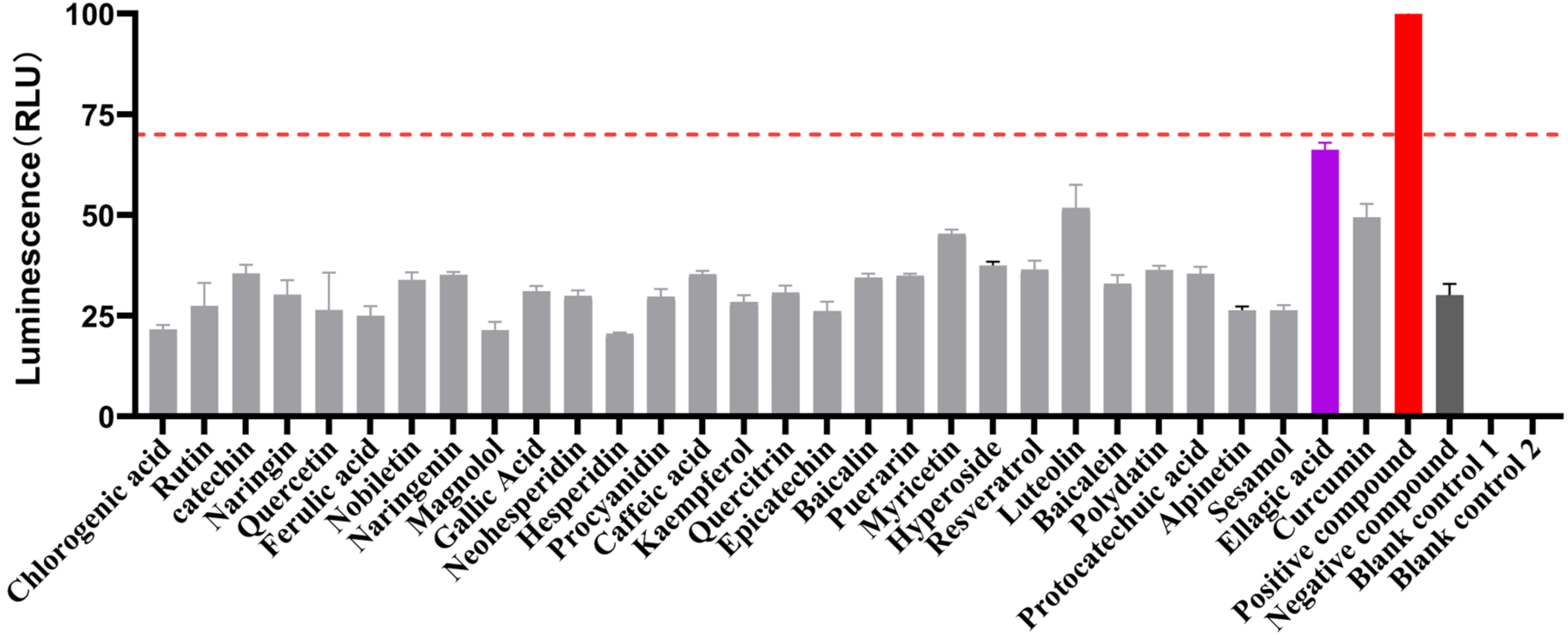

Table S1. 30 Polyphenolic compounds

Name	Biological activity	Source	Structure
Chlorogenic acid	Anti-inflammatory, antioxidant, antitumor, antimicrobial, and neuroprotective	Honeysuckle, Coffee, Chrysanthemum	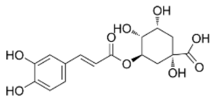
Rutin	Antioxidant, anti-inflammatory, anti-tumor, hypoglycemic, neuroprotective, anti-bacterial, anti-aging	SophorajaponicaL, Jujube, hawthorn, and ginkgo	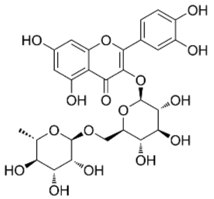
Catechin	Antitumor, antioxidant and antibacterial	Tea, apples, persimmons, cacaos, grapes, and berries	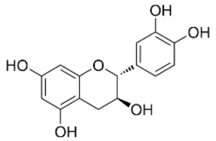
Naringin	P450 enzymes inhibito; Antioxidant and anticancer	Citrus fruits	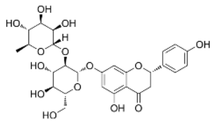
Quercetin	heat shock protein inhibitor, PI3K inhibitor	Onion, black tea, tomato, apple, nut	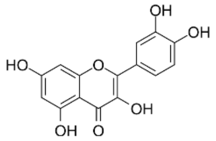

Ferulic acid	FGFR1 inhibitor	Coffee, wheat bran, Angelica, Ligusticum, Cimicifuga, Sour Jujube Seed	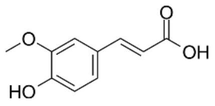
Nobiletin	Anti-inflammation, anti-cancer, anti-angiogenesis, anti-proliferation, anti-metastasis	Citrus peel	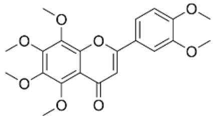
Naringenin	Strong anti-inflammatory and antioxidant	Citrus	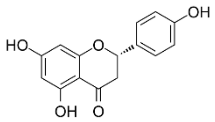
Magnolol	Agonist of both RXRα and PPARγ	the stem bark of Magnolia officinalis	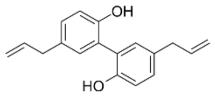
Gallic Acid	Antimicrobial, antioxidant, antimicrobial, anti-inflammatory, and anticance	Tea leaves, nuts, strawberries, blueberries, black beans	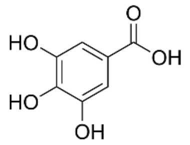
Neohesperidin	Antioxidant and anti-inflammatory	Citrus plants	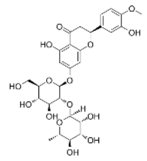

Hesperidin	Antioxidant, antitumor and antiallergic	Citrus fruits	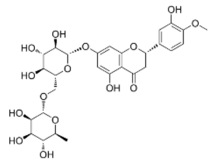
Apigenin	Anti-inflammatory and antioxidant;	Celery, garlic, broccoli, onion, apple, orange	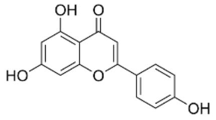
Caffeic acid	TRPV1 and 5-Lipoxygenase inhibitor	Apples, grapes, coffee beans	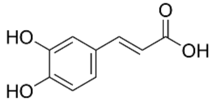
Kaempferol	Antitumor, anti-inflammation, antioxidant, antibacterial and antiviral	Tea leaves, broccoli, grapes, apples	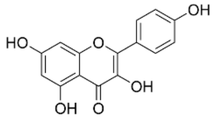
Quercitrin	anti-inflammation, antioxidative and neuroprotective	Celery, onion, apple	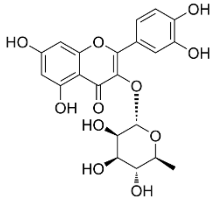
Epicatechin	Antioxidant, lipid-lowering and blood sugar-lowering, cardiovascular disease prevention, anti-inflammatory, neuroprotective, antibacterial	Lychee, propolis, grape seed, red wine	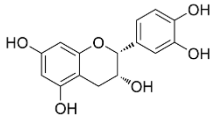

Baicalin	antioxidant, anti-tumor, anti-HIV	Scutellaria baicalensis	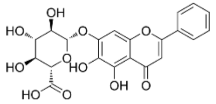
Puerarin	5-HT2C antagonist	Kudzu Root	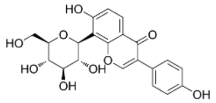
Myricetin	strong anti-oxidant, anticancer, antidiabetic and anti-inflammatory	Honey, bayberry, grapes	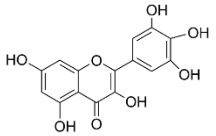
Hyperoside	Anti-tumor, antifungal, anti-inflammatory, anti-viral, and anti-oxidative	Hypericum, Hawthorn	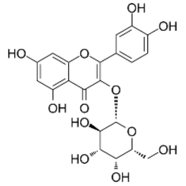
Resveratrol	Anti-oxidant, anti-inflammatory, cardioprotective, and anti-cancer	Grapes, peanuts, blueberries	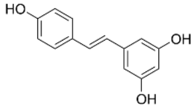
Luteolin	Nrf2 inhibitor; anti-inflammatory and anti-cancer	Perilla, celery, broccoli	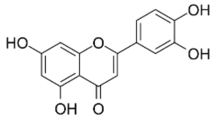
Baicalein	CYP2C9 inhibitor; antitumor, anti-inflammatory, and antibacterial	Scutellaria baicalensis	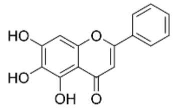

Ellagic acid	CK2 inhibitor; antioxidant	Blueberries, pomegranates, grapes, nuts	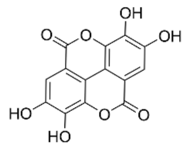
Curcumin	NF-κB inhibitor; anti-inflammatory, antioxidant, antiproliferative and antiangiogenic	Curcuma longa	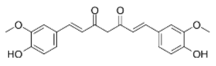
Alpinetin	antitumor, antiinflammation, antibacterial, antiviral, neuroprotective	Turmeric、 Alpinia katsumadai	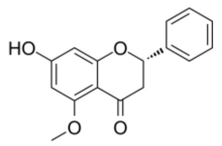
Sesamol	Anti-oxidant and anticancer	Sesame	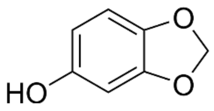
Polydatin	Antioxidant, anti-tumor, anti-inflammatory, neuroprotective, cardioprotective	Japanese knotweed, Grapes, peanuts, mulberries	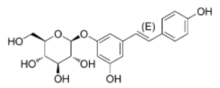
Protocatechuic acid	Antibacterial and neuroprotective effect	Garlic, Cinnamon, Turmeric, Ginkgo	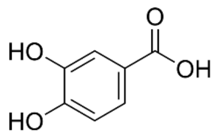

Table S2. Disease activity index（DAI）

Score	Weight loss	Fecal consistency	Fecal occult blood
0	None	Normal	Negative
1	1-5%	Soft stool	Light Blue
2	5-10%	Mucoid stools	Blue+
3	10-20%	Diarrhea	Dark Blue
4	>20%		Gross bleeding

Table S3. Histopathological Score

Score	Inflammation severity	Inflammation extent	Crypt damage	Range of lesions (%)
**0**	None	None	None	0
**1**	Mild	Mucosa	Basal 1/3 damage	1-25
**2**	Moderate	Submucosa	Basal 2/3 damage	26-50
**3**	Severe	Transmural	Crypt lost, surface epithelium present	51-75
**4**	-	-	Crypt and surface epithelium lost	76-100
